# Intranasal administration of cationic liposomes enhanced granulocyte–macrophage colony-stimulating factor expression and this expression is dispensable for mucosal adjuvant activity

**DOI:** 10.1186/s13104-018-3591-3

**Published:** 2018-07-13

**Authors:** Rui Tada, Akira Hidaka, Hiroshi Kiyono, Jun Kunisawa, Yukihiko Aramaki

**Affiliations:** 1 Department of Drug Delivery and Molecular Biopharmaceutics, School of Pharmacy, Tokyo University of Pharmacy and Life Sciences, 1432-1, Horinouchi, Hachioji, Tokyo 192-0392 Japan; 2 Division of Mucosal Immunology and International Research and Development Center for Mucosal Vaccines, Department of Microbiology and Immunology, The Institute of Medical Science, The University of Tokyo, Tokyo, Japan; 3 Laboratory of Vaccine Materials, National Institutes of Biomedical Innovation, Health and Nutrition (NIBIOHN), Osaka, Japan

**Keywords:** Cationic liposome, Granulocyte–macrophage colony-stimulating factor, Intranasal immunization, Mucosal adjuvant

## Abstract

**Objective:**

Infectious diseases remain a threat to human life. Vaccination against pathogenic microbes is a primary method of treatment as well as prevention of infectious diseases. Particularly mucosal vaccination is a promising approach to fight against most infectious diseases, because mucosal surfaces are a major point of entry for most pathogens. We recently developed an effective mucosal adjuvant of cationic liposomes composed of 1,2-dioleoyl-3-trimethylammonium-propane (DOTAP) and 3β-[*N*-(*N*′,*N*′-dimethylaminoethane)-carbamoyl] (DC-chol) (DOTAP/DC-chol liposomes). However, the mechanism(s) underlying the mucosal adjuvant effects exerted by the cationic liposomes have been unclear. In this study, we investigated the role of granulocyte–macrophage colony-stimulating factor (GM-CSF), which was reported to act as a mucosal adjuvant, on the mucosal adjuvant activities of DOTAP/DC-chol liposomes when administered intranasally to mice.

**Results:**

Here, we show that, although intranasal vaccination with cationic liposomes in combination with antigenic protein elicited GM-CSF expression at the site of administration, blocking GM-CSF function by using an anti-GM-CSF neutralizing antibody did not alter antigen-specific antibody production induced by DOTAP/DC-chol liposomes, indicating that GM-CSF may not contribute to the mucosal adjuvant activity of the cationic liposomes when administered intranasally.

**Electronic supplementary material:**

The online version of this article (10.1186/s13104-018-3591-3) contains supplementary material, which is available to authorized users.

## Introduction

Vaccines have been a great public health success in past decades. However, the development of additional safe and efficient vaccines against various infectious diseases is still a challenge [1–3]. Among vaccine development approaches, mucosal vaccines are most attractive for treating/preventing infectious diseases caused by pathogenic microbes, because most pathogens enter the host body via mucosal surfaces [4, 5]. However, the addition of mucosal adjuvants to mucosal vaccine formulations is crucial to inducing antigen-specific immune responses to proteins derived from microbes, because these antigens show poor immunogenicity in mucosal compartments [6].

We have recently found that nasal immunization of an antigenic protein with cationic liposomes composed of 1,2-dioleoyl-3-trimethylammonium-propane (DOTAP) and 3β-[*N*-(*N*′,*N*′-dimethylaminoethane)-carbamoyl] (DC-chol) (DOTAP/DC-chol liposomes) potently induced both mucosal and systemic immune responses to the antigen in mice [7–9]. Although the molecular mechanisms underlying the mucosal adjuvant effects exerted by cationic liposomes was unclear, we revealed that these cationic liposomes promote the uptake of antigenic proteins by dendritic cells (DCs) in nasal-associated lymphoid tissues (NALTs) in vivo. In general, one of the molecular mechanisms of adjuvants is increased uptake of antigen and presentation to major histocompatibility complex (MHC) class II on antigen-presenting cells (APCs). However, activation of innate immunity might be much more important for their adjuvant activities, including recruitment of innate immune cells at the site of administration and induction of cytokines and chemokines [10–12]. These views led us to investigate the role of cytokines in the mucosal adjuvant activity of DOTAP/DC-chol liposomes.

In the present study, we hypothesized that granulocyte–macrophage colony-stimulating factor (GM-CSF) might be associated with the mucosal adjuvant effects of DOTAP/DC-chol liposomes administered intranasally to mice. GM-CSF is involved in various biological phenomena, such as promoting cell differentiation, activation, survival, and induction of inflammatory responses [13, 14], as well as inducing mucosal and serum antibody responses when co-administered with antigenic proteins via the nasal route in mice [15–18]. Thus, in this study, we examined the function of GM-CSF in DOTAP-DC-chol liposome-induced antigen-specific antibody responses in both mucosal and systemic area in mice.

## Main text

### Methods

#### Mice and materials

Female BALB/cCrSlc mice (7–10 weeks old) were purchased from Japan SLC (Hamamatsu, Shizuoka, Japan). Animals were housed in a specific pathogen-free environment and all animal experiments were approved by the institution’s committee for laboratory animal experiments of the Tokyo University of Pharmacy and Life Sciences (P13–22, P14–31, and P15–33). 1,2-Dioleoyl-3-trimethylammonium-propane (DOTAP) and 3β-[*N*-(*N*′,*N*′-dimethylaminoethane)-carbamoyl] (DC-chol) were purchased from Avanti Polar Lipids (Alabaster, AL, USA). Recombinant mouse GM-CSF, anti-GM-CSF neutralizing antibody, and rat IgG2a κ isotype control antibody were all purchased from BioLegend (San Diego, CA, USA). Low endotoxin (less than 1 EU/mg, guaranteed) egg white ovalbumin (OVA) was obtained from Wako Pure Chemical Industries (Osaka, Japan).

#### Preparation of liposomes

DOTAP/DC-chol liposomes were prepared as follow [7]. 10 μmol of total lipid dissolved in chloroform (DOTAP:DC-chol at a 1:1 mol ratio) was evaporated to dryness to obtain the lipid films. The lipid films were then hydrated in 250 μL of phosphate-buffered saline (PBS) and vortexed for 5 min. The prepared liposomes were extruded 10 times by passage through an appropriate pore size polycarbonate membrane (Advantec, Tokyo, Japan) and sterilized via filtration (0.45-μm filter membranes; Iwaki, Tokyo, Japan).

#### Immunization schedule

Mice were immunized twice intranasally once a week (days 0 and 7). Mice were divided into three groups as follows: (1) vehicle (PBS), (2) OVA alone (5 µg/mouse), or (3) OVA (5 µg/mouse) plus liposomes (400 nmol/mouse) or recombinant mouse GM-CSF (4 µg/mouse described previously [19]). After sacrificing the mice by sodium pentobarbital administration (100 mg/kg body weight, intraperitoneal), serum and nasal wash samples were collected on day 14, as described previously [20, 21].

#### Detection of OVA-specific antibody

A 96-well plate was coated with OVA in a 0.1 M carbonate buffer (pH 9.5). The plate was washed and then blocked with 1% bovine serum albumin (BSA; Wako Pure Chemical Industries) containing PBST (BPBST) for 60 min at 37 °C. After washing, the plate was incubated with samples (serum or nasal wash) for 60 min at 37 °C. To detect OVA-specific IgG antibody, plates were washed with PBST, treated with peroxidase-conjugated anti-mouse IgG (Sigma-Aldrich, St. Louis, MO, USA) in BPBST. The plate was washed, and combined with TMB substrate (KPL, Maryland, USA) and then further incubated for color development. To detect OVA-specific IgA, IgG1, and IgG2a, plates were treated with biotin-conjugated anti-mouse IgA, IgG1, or IgG2a (BioLegend) in BPBST, and then avidin-horseradish peroxidase (BioLegend) in PBST was added. Plates were incubated with TMB substrate system (KPL). The reaction was terminated with 1 N phosphoric acid, and optical densities were measured at 450 nm/650 nm [22–24]. Endpoint titers were calculated as the reciprocal of the last dilution exceeding a cut-off value that was twice the mean of a negative control [25, 26].

#### Preparation of splenocytes for cell culture

Splenocytes were prepared as described earlier [13, 27]. Briefly, after sacrificing the mice by cervical dislocation, their spleens were excised and dissociated in RPMI 1640 medium (Wako Pure Chemical Industries). The resulting single-cell suspension was then treated with ACK lysis buffer (BioLegend). After centrifugation, splenocytes were suspended in RPMI 1640 medium supplemented with 10% heat-inactivated fetal bovine serum (FBS; Biowest, Nuaillé, France), 100 U/mL of penicillin G potassium salt (Sigma-Aldrich), and 100 μg/mL of streptomycin sulfate salt (Sigma-Aldrich). The cells were cultured at 2 × 10^6^ cells/well in 0.5 mL of culture medium in 48-well flat-bottomed plates (IWAKI) and re-stimulated with OVA (Wako Pure Chemical Industries) for the indicated time at 37 °C in a 5% CO_2_.

#### Cytokine assay

The cytokine concentrations were measured using ELISA MAX Standard Sets (BioLegend) according to the manufacturer’s instructions. The data were expressed as the mean ± standard deviation. At least three independent experiments were conducted.

#### RNA extraction and quantitative real time-polymerase chain reaction (qPCR)

BALB/c female mice were euthanized by sodium pentobarbital administration (100 mg/kg body weight, intraperitoneal). Their nasal tissues and spleens were then excised, and the total RNA was extracted from these samples using a FavorPrep Tissue Total RNA Mini Kit (Favorgen Biotech Corporation, Ping-Tung, Taiwan), followed by DNase I (Roche Life Science, Penzberg, Germany) treatment. cDNA was synthesized from total RNA using a ReverTra Ace qPCR RT Master Mix (Toyobo, Tokyo, Japan). Then, qPCR was carried out according to the manufacturer’s instructions using a THUNDERBIRD SYBR qPCR Mix (Toyobo). The primers used for PCR were the following: GM-CSF, forward, 5′-TGGGCATTGTGGTCTACAGC-3′, and reverse, 5′-GCGGGTCTGCACACATGTTA-3′; β2-microglobulin, forward, 5′-TTCTGGTGCTTGTCTCACTGA-3′, and reverse, 5′-CAGTATGTTCGGCTTCCCATTC-3′. The expression of GM-CSF was determined using the comparative *Δ*-threshold cycle method using β2-microglobulin as a reference gene. GM-CSF expression is presented as the fold change relative to expression in the control sample.

#### Effect of anti-GM-CSF neutralizing antibody on mucosal adjuvant activity

BALB/c female mice were pre-treated intraperitoneally with anti-GM-CSF neutralizing antibody (100 µg/mouse) 2 days before (day-2) and 1 h before immunization (days 0 and 7) as reported previously [28–31]. Mice were divided into three groups as follows: (1) PBS, (2) OVA alone (5 µg/mouse), or (3) OVA (5 µg/mouse) in combination with liposomes (400 nmol/mouse) on days 0 and 7. After sacrificing the mice by sodium pentobarbital administration (100 mg/kg body weight, intraperitoneal), serum and nasal wash samples were collected on day 14, as described previously [20, 21].

#### Statistics

Statistical differences were calculated with unpaired *t*-test with Welch’s correction and the Kruskal–Wallis test with Dunn’s post hoc test for cytokine and antibody production, respectively. Differences with *p* values of < 0.05 were considered significant.

### Results

#### Antigen-specific nasal and serum antibodies induced by intranasal immunization of OVA with the cationic liposomes

First, we evaluated the production of OVA-specific antibodies after intranasal immunization of OVA in combination with DOTAP/DC-chol liposomes in BALB/c female mice. As expected, intranasal vaccination induced the production of OVA-specific nasal IgA in nasal fluid and IgG in the serum compartment. In contrast, intranasal immunization with PBS (vehicle) or OVA alone did not exhibit significant OVA-specific antibody production in either mucosal or systemic under these experimental conditions (Additional file 1: Figure S1).

#### Expression of GM-CSF in mucosal and systemic sites of vaccinated mice

**Fig. 1:**
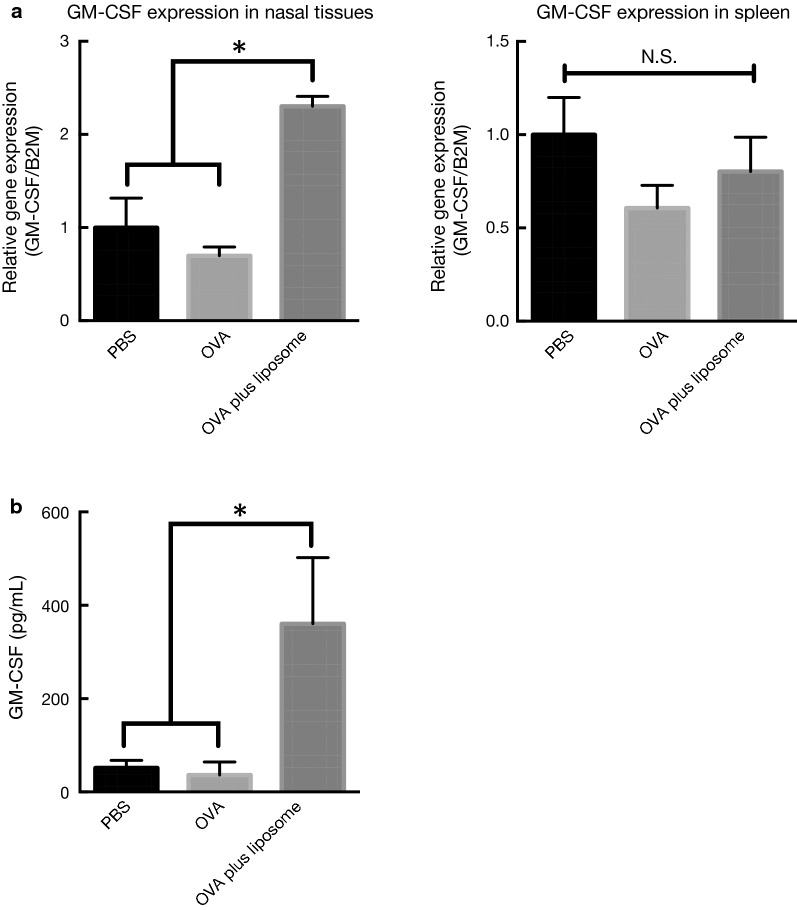
mRNA expression of granulocyte–macrophage colony-stimulating factor (GM-CSF) in nasal and spleen tissues (**a**) and antigen-specific production of GM-CSF in splenocytes from mice administered intranasally with OVA plus the liposomes. **a** Nasal tissue and spleens were collected 6 h after the last immunization. mRNA expression was measured using qPCR. **b** Spleens were collected 1 week after the last immunization, and then harvested splenocytes were cultured for 72 h in the presence of OVA (10 μg/mL). After culture, concentrations of GM-CSF were determined using ELISA. The values are the mean ± SD of technical duplicates from three biologically independent experiments. Significance was assessed using unpaired *t*-test with Welch’s correction; *p < 0.05

Prior to assessing the contribution of GM-CSF on the mucosal adjuvant activities of the cationic liposomes, we first examined the expression of GM-CSF at the site of injections. As shown in Fig. 1a, intranasal administration with OVA and DOTAP/DC-chol liposomes significantly exerted the expression of GM-CSF in nasal areas (2.3- to 3.3-fold expression compared to mice that received either vehicle or OVA alone). On the other hand, immunization with OVA and DOTAP/DC-chol liposomes did not induce any GM-CSF expression in the spleen. Additionally, since it is known that T cells activated via TCR signalling are capable of producing a large amount of GM-CSF [32, 33], we investigated antigen-specific GM-CSF secretion in splenocytes by re-stimulating them with OVA in vitro. As shown in Fig. 1b, splenocytes from OVA and DOTAP/DC-chol liposome-vaccinated mice produced higher levels of GM-CSF than those from OVA-only administered mice when re-stimulated with OVA. These results indicated that nasally administered DOTAP/DC-chol liposomes were polarized to induce the expression of GM-CSF.

#### Effect of GM-CSF on the mucosal adjuvant activities of nasally administered cationic liposomes

**Fig. 2:**
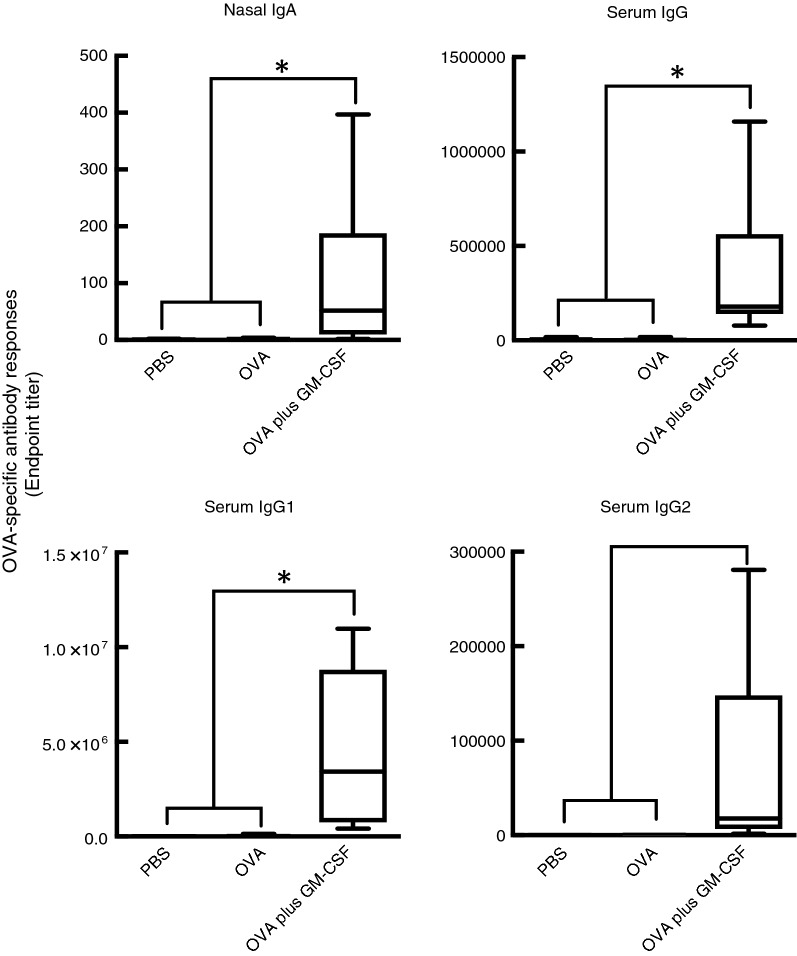
Recombinant GM-CSF induces both mucosal and systemic OVA-specific antibody responses. The data show the OVA-specific nasal IgA and serum IgGs for each immunized group (PBS only, OVA alone, or OVA plus recombinant GM-CSF). The data were obtained from three independent experiments. The statistically significant value (*p < 0.0001) shown were calculated from the Kruskal–Wallis test with Dunn’s post hoc test

**Fig. 3:**
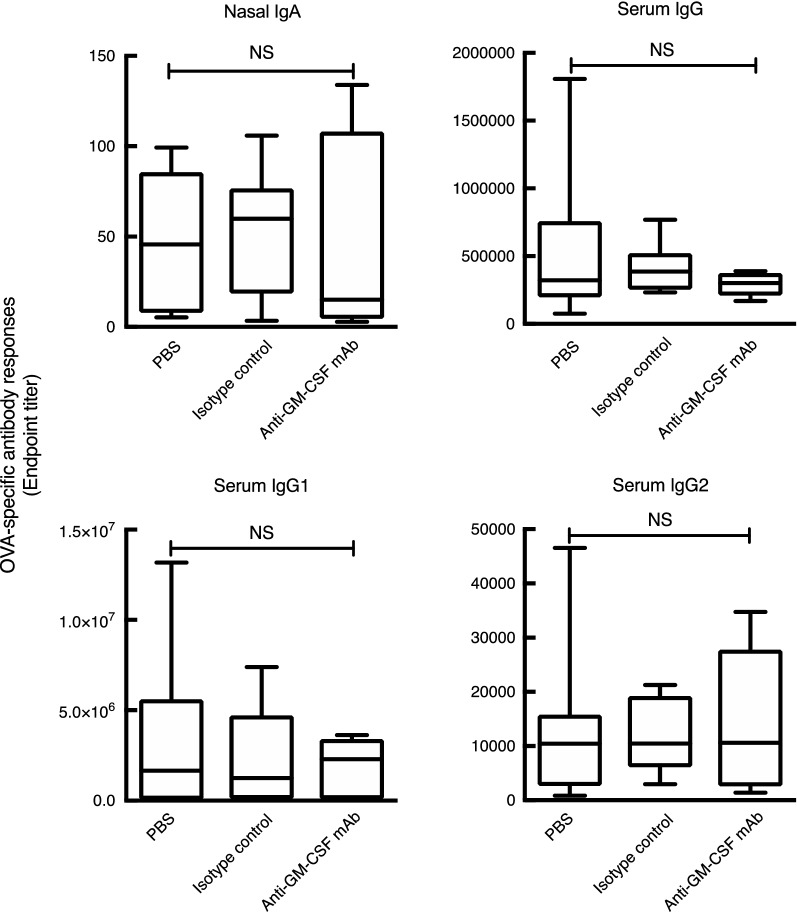
Effect of anti-GM-CSF neutralizing antibodies on ovalbumin (OVA)-specific antibody responses induced by DOTAP/DC-chol liposomes. Mice were pre-treated intraperitoneally with anti-GM-CSF neutralizing antibody (100 µg/mouse) at day 0 and then immunized intranasally with PBS, OVA alone, or OVA plus DOTAP/DC-chol liposomes on days 2 and 9. Nasal washes and sera were collected on day 16. OVA-specific nasal IgA and serum IgGs were detected using ELISA. The data were obtained from three independent experiments. *NS* not significant as evaluated using the Kruskal–Wallis test with Dunn’s post hoc test

We next explored the role of GM-CSF in the effects of DOTAP/DC-chol liposomes as a mucosal adjuvant when administered intranasally. Before exploring this association, the mucosal adjuvant effect of nasally administered recombinant GM-CSF was examined. As expected, intranasal administration with OVA and recombinant GM-CSF induced the production of OVA-specific IgA in nasal fluid and IgG in serum samples (Fig. 2), which are almost same that of DOTAP/DC-chol liposomes alone (Additional file 1: Figure S1). Next, we examined whether the induction of mucosal adjuvant effects by DOTAP/DC-chol liposomes were dependent on GM-CSF using an anti-GM-CSF neutralizing monoclonal antibody (mAb) as previously described to block the biological activities of GM-CSF in vivo [28–31]. Pre-treatment with anti-GM-CSF neutralizing antibody did not affect nasal IgA or serum IgG, IgG1, or IgG2 antibody production, showing that GM-CSF expression was not required for the mucosal adjuvant activities of cationic liposomes (Fig. 3).

### Discussion

In this study, we demonstrated the following: (1) intranasal administration with DOTAP/DC-chol liposomes induced the expression of GM-CSF at the site of injections; (2) recombinant GM-CSF showed the mucosal adjuvant effect when nasally administered in mice; and (3) GM-CSF expression in nasal area induced by DOTAP/DC-chol liposomes was not required for the mucosal adjuvant activities.

The development of safe and efficient mucosal adjuvants is needed to prevent fatal infectious diseases. To accomplish this, understanding the mechanism(s) underlying mucosal adjuvant induction of immune responses to antigenic proteins is essential. Generally, adjuvants show their activities through the depot effect, with the gradual release of antigen at the site of infection and increase in antigen uptake by APCs. Resent research has focused on the role of APCs in activating innate immunity [34]. In particular, the cytokine/chemokine milieu induced by external stimuli, including adjuvants, determines the immune response to antigenic proteins, including the production of antibody to the antigen [35–37]. Many studies on the immunomodulating activities of GM-CSF have been reported. For instance, GM-CSF has been shown to stimulate the maturation and function of APCs, such as DCs and macrophages. GM-CSF is also a strong inducer of interleukin-6 (IL-6), which promotes germinal center development and B cell growth and differentiation in these centers [38, 39]. We found that intranasal administration of DOTAP/DC-chol liposomes induced IL-6 expression in the nasal mucosa, and that this cytokine was critical for the induction of antigen-specific IgA by the cationic liposomes (unpublished results). Therefore, local GM-CSF expression likely plays a role in enhancing humoral responses to the cationic liposomes. Furthermore, intranasal co-administration of antigens with a GM-CSF-expressing plasmid has been shown to increase OVA-specific mucosal IgA and serum IgG titers, suggesting that GM-CSF plays an essential role in the induction of humoral immune responses to antigenic proteins in both mucosal and systemic compartments [10, 18, 19, 40]. We investigated the role of GM-CSF on the mucosal adjuvant activities of the cationic liposomes in this study and found that GM-CSF blocking did not affect their activities, clearly indicating that other soluble factors control the mucosal adjuvant activities of the cationic liposomes. Further experiments are required to clarify the molecular mechanism(s) underlying the induction of humoral immune responses by the cationic liposomes.

## Limitations

Herein, we demonstrated that nasal administration of DOTAP/DC-chol liposomes induced the gene expression of GM-CSF at the site of administration; however, the protein level of GM-CSF in the nasal area after the nasal immunization of DOTAP/DC-chol liposomes has not been evaluated. The major limitation of this study is that though the protocol for studying the biological activities of GM-CSF neutralization at the mucosal compartments by intraperitoneal injection of anti-GM-CSF neutralizing antibody has been reported in literatures [28–31], we have not confirmed these neutralizing effects in this study. Overall, although our data suggested that GM-CSF may not be required for the mucosal adjuvant effects of the cationic liposomes, we were unable to identify the possible mechanism(s) for these effects in this study. Further experiments are required in the future to clarify these aspects.

## Additional file

**Additional file 1: Figure S1.** DOTAP/DC-chol liposomes potentiate both mucosal and systemic OVA-specific antibody responses. The data show the OVA-specific nasal IgA and serum IgGs for each immunized group (PBS only, OVA alone, or OVA plus liposomes). The data were obtained from three independent experiments. The statistically significant value (*p < 0.0001) shown were calculated from the Kruskal–Wallis test with Dunn’s post hoc test.

## Abbreviations

APCs: antigen-presenting cells
DC-chol: 3β-[*N*-(*N*′,*N*′-dimethylaminoethane)-carbamoyl]
DCs: dendritic cells
DOTAP: 1,2-dioleoyl-3-trimethylammonium-propane
ELISA: enzyme linked immunosorbent assay
GM-CSF: granulocyte–macrophage colony-stimulating factor
IL-6: interleukin-6
MHC: major histocompatibility complex
NALTs: nasal-associated lymphoid tissues
OVA: ovalbumin from egg white
PBS: phosphate-buffered saline
qPCR: quantitative PCR
TMB: tetramethylbenzidine
TSLP: thymic stromal lymphopoietin
