# Supplementary material for: Intranasal administration of cationic liposomes enhanced granulocyte–macrophage colony-stimulating factor expression and this expression is dispensable for mucosal adjuvant activity
Source: BMC Res Notes. 2018 Jul 13;11:472. doi: 10.1186/s13104-018-3591-3 (PMC6045820; doi:10.1186/s13104-018-3591-3)
Supplement: Supplementary file 1 — Additional file 1: Figure S1. DOTAP/DC-chol liposomes potentiate both mucosal and systemic OVA-specific antibody responses. The data show the OVA-specific nasal IgA and serum IgGs for each immunized group (PBS only, OVA alone, or OVA plus liposomes). The data were obtained from three independent experiments. The statistically significant value (*p < 0.0001) shown were calculated from the Kruskal–Wallis test with Dunn’s post hoc test. [file 13104_2018_3591_MOESM1_ESM.pdf]

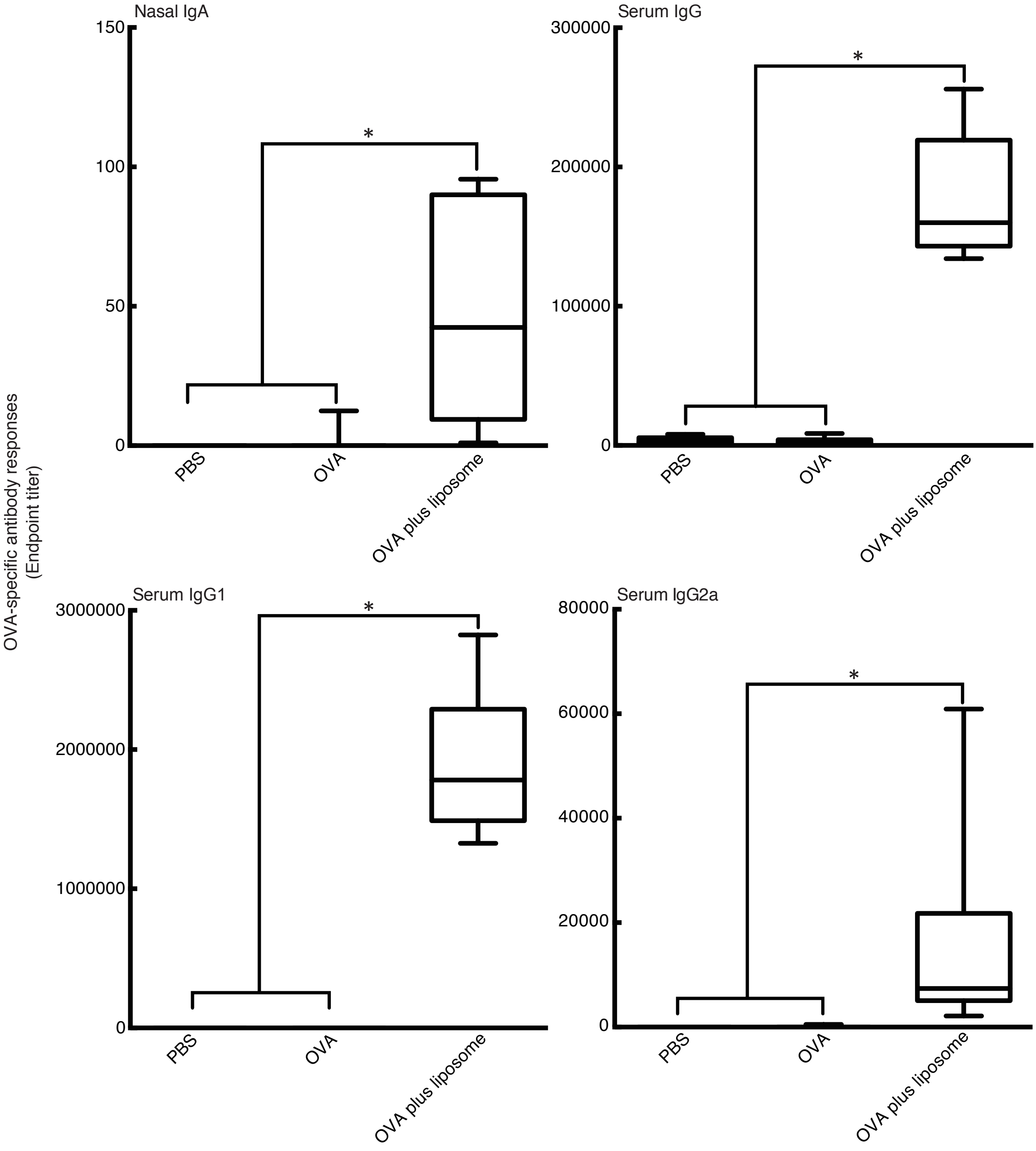

Additional file 1: Figure S1. Tada et al.

DOTAP/DC-chol liposomes potentiate both mucosal and systemic OVA-specific antibody responses. The data show the OVA-specific nasal IgA and serum IgGs for each immunized group (PBS only, OVA alone, or OVA plus liposomes). The data were obtained from three independent experiments. The statistically significant value (**p*<0.0001) shown were calculated from the Kruskal-Wallis test with Dunns post-hoc test.
